# Pelvic peritonitis during biologic therapy for rheumatoid arthritis: a case report and review of the literature

**DOI:** 10.1186/2193-1801-3-567

**Published:** 2014-09-29

**Authors:** Tsuyoshi Sasaki, Koichi Okamura, Yukio Yonemoto, Chisa Okura, Kenji Takagishi

**Affiliations:** Department of Orthopaedic Surgery, Gunma University Graduate School of Medicine, 3-39-22, Showamachi, Maebashi, Gunma 371-8511 Japan

**Keywords:** Biologics, Methotrexate, Pelvic peritonitis, Rheumatoid arthritis, Tacrolimus

## Abstract

**Introduction:**

Infections are recognized as major complications during therapy with biologics and other immunosuppressant drugs. The respiratory tract, bone, joint, skin, and soft tissues are well known sites of infection in patients with rheumatoid arthritis (RA) treated by biologics or other immunosuppressants. It is known that patients with intra-abdominal infections may develop tuberculous peritonitis during biologic therapy. However, non-tuberculous pelvic peritonitis is rare.

**Case description:**

A case of a 46-year-old patient with RA developed pelvic peritonitis during therapy with MTX, tacrolimus (TAC), and golimumab (GLM). The patient visited our hospital due to a fever and general malaise. Physical findings included lower abdominal tenderness and rebound tenderness. Abdominal computed tomography (CT) images showed an intrauterine foreign body and ascites. The contraceptive ring was removed. Streptococcus agalactiae and Streptococcus constellatus were cultured from the removed contraceptive ring. She was started on an antimicrobial agent, flomoxef (FMOX), at 2 g/day. The FMOX dosage was increased to 3 g/day from the 3rd day of disease and continued for 10 days. Her fever disappeared from the 4th disease day, and her inflammatory response then gradually decreased. No exacerbation of symptoms occurred even after the FMOX treatment was stopped, and the patient was discharged on the 14th disease day.

**Discussion and evaluation:**

MTX and biologics were being administered at the time of onset of peritonitis. The peritonitis was diagnosed on the basis of the gynecological evaluation and CT imaging findings that were typical of peritonitis. The patient was in an immunosuppressed state during administration of anti-rheumatic drugs, and the peritonitis was thought to have developed due to an ascending infection via the long-term presence of the intrauterine contraceptive ring which had an attached string.

**Conclusions:**

Before starting biological agents, patients must be questioned regarding the presence of an intrauterine foreign body.

## Background

Many different adverse events (AEs), including infections, interstitial pneumonia, and infusion-related reactions, can occur during administration of methotrexate (MTX) and/or biologics. Of these AEs, infections require special attention (Favalli et al. 2009; Conway et al. 2014). Most infections associated with rheumatoid arthritis (RA) are respiratory infections (Doran et al. 2002), though RA patients are reported to be at higher risk than the general population especially for infections of the bones and joints, as well as the skin and soft tissues (Doran et al. 2002). It is known that patients with intra-abdominal infections may develop tuberculous peritonitis during biologic therapy (Verhave et al. 2008), but there are no reports of infections of the genitalia or adnexa.

In this report, the case of a 46-year-old patient with RA who developed pelvic peritonitis during therapy with MTX, tacrolimus (TAC), and golimumab (GLM) is described.

## Case presentation

The patient was a 46-year-old woman with a history of myasthenia gravis who presented with chief complaints of fever and general malaise. The patient was diagnosed as having RA in 2006, and she was treated with methotrexate (MTX). Because of insufficient efficacy, adalimumab (ADA) was added in 2010. Because of secondary failure, ADA was switched to golimumab (GLM), beginning at 50 mg/4 weeks in 2012. Although the disease activity remained high even after the dosage of GLM was increased to 100 mg/4 weeks, tacrolimus (TAC) was administered concomitantly. During these periods, the RA disease activity ranged from moderate to high. The patient visited our hospital in June 2013 due to a fever and general malaise. Vital signs at presentation were temperature 38.6°C, blood pressure 113/70 mmHg, pulse 87/min, and SpO_2_ 97% (room air). Physical findings included lower abdominal tenderness and rebound tenderness.

Blood tests showed a WBC count of 15,300/μL and CRP of 13.25 mg/dL, indicating an elevated inflammatory response. Table 1 lists the results of the blood tests at presentation.

**Figure 1:**
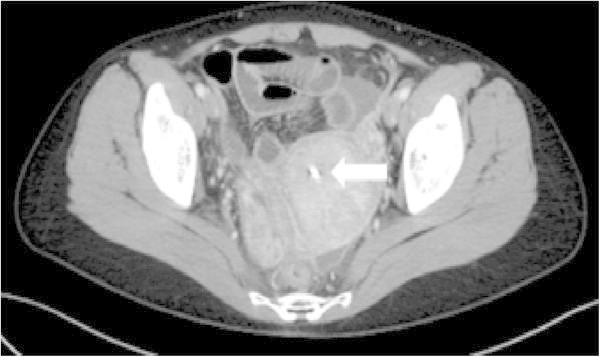
**Plain CT of the pelvic region.** White arrow: Intrauterine foreign body.

Abdominal computed tomography (CT) images showed an intrauterine foreign body and ascites. High-density peritoneal adipose tissue and peritoneal thickening were also observed (Figure 1).

**Figure 2:**
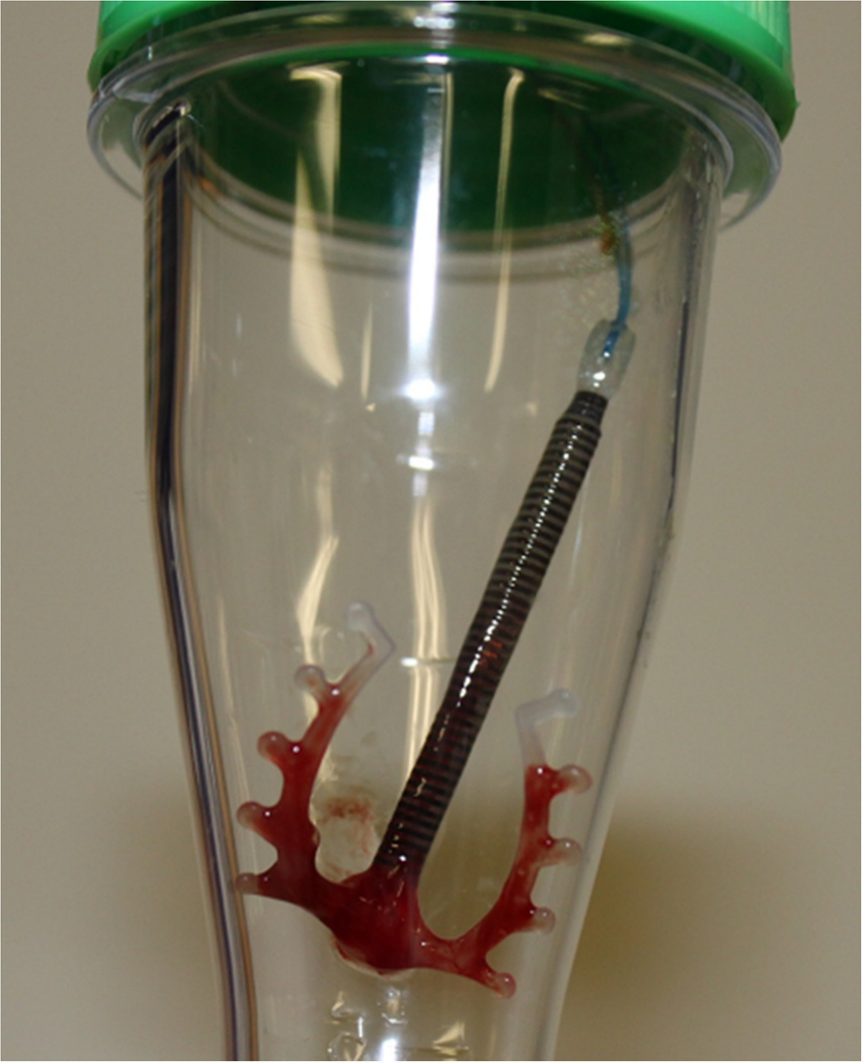
**Removed contraceptive ring.**

Based on a gynecological examination, the patient was diagnosed as having pelvic peritonitis due to an intrauterine infection associated with a contraceptive ring and an ascending infection. The contraceptive ring was removed (Figure 2). The patient was on anti-rheumatic drugs, which were all discontinued. She was started on an antimicrobial agent, flomoxef (FMOX), at 2 g/day. The FMOX dosage was increased to 3 g/day from the 3rd day of disease and cont for 10 days. Her fever disappeared from the 4th day, and her inflammatory response then gradually decreased. No exacerbation of symptoms occurred even after the FMOX treatment was stopped, and the patient was discharged on the 14th day.

**Table 1:** **Blood test results at presentation**

WBC	15,300/μL	Alb	3.6 g/dL
Neutrophils	87.8%	T-Bil	0.5 mg/dL
Lymphocytes	8.4%	AST	36 U/L
RBC	4.57 × 10^6^/μL	ALT	25 U/L
Hb	13.1 g/dL	LDH	210 U/L
Plt	307 × 10^3^/μL	BUN	15 mg/dL
CRP	13.25 mg/dL	Cr	0.54 mg/dL
Na	138 mEq/L	ESR	76 mm/h
K	3.8 mEq/L	MMP-3	86.3 ng/mL
Cl	104 mEq/L		

*ESR* Erythrocyte sedimentation rate, *MMP-3* Matrix metalloproteinase-3.

*Streptococcus agalactiae* (group G beta-hemolytic streptococcus) and *Streptococcus constellatus* were cultured from the removed contraceptive ring, while blood cultures were negative.

Following the onset of the peritonitis, the joint swelling and tenderness improved during treatment. However, the RA symptoms relapsed 2 months after the onset of the peritonitis. Therefore, administration of GLM + MTX + TAC was re-started at that time, after which there were no signs of infection.

## Discussion

This patient had been diagnosed with RA 7 years earlier. MTX and biologics were being administered at the time of onset of peritonitis. The peritonitis was diagnosed on the basis of the gynecological evaluation and CT imaging findings that were typical of peritonitis. The patient was in an immunosuppressed state during administration of anti-rheumatic drugs, and the peritonitis was thought to have developed due to an ascending infection via the long-term presence of the intrauterine contraceptive ring that had an attached string.

Pelvic peritonitis is an ascending infection that is introduced via the uterus (Centers for Disease control and Prevention 1997; Svalingam et al. 2007; Khan & Rizvi 2006). The pathogenic bacteria are not limited to just chlamydia and gonococci, which are usually sexually transmitted (Svalingam et al. 2007), but they include general aerobic and anaerobic bacteria that are resident in the cervix and vagina. Mixed infections with these bacteria are also common (Khan & Rizvi 2006; Kielly & Jamieson 2014). Group G beta-hemolytic streptococci are bacteria that reside in the lower gastrointestinal tract, female urinary tract, and genitalia (Heath & Jardin 2014), and caution is essential in the case of patients, as in the present case, who are on immunosuppressive agents.

Insertion of contraceptive rings has been reported to cause an increased incidence of intrapelvic infections (Elhag et al. 1988; Mead et al. 1975; Kaliterna et al. 2011). Moreover, since the risk of infection increases as a result of the long-term presence of a contraceptive ring, there are reports recommending that replacement be performed after 5 years of use (Pal et al. 2005). In particular, intrauterine contraceptive rings with an attached string have been found to readily cause ascending infections via the string that is continuous from within the uterus to the vagina (Batar et al. 1988), and caution is thus required.

The present patient also had a contraceptive ring with an attached string that had been in place long-term, and it was considered to be the cause of the infection. Moreover, this patient had forgotten that she had a contraceptive ring. When administration of biologics or immunosuppressive agents is started, it is necessary for the physician to thoroughly interview the patients’ life history, not just the underlying disease.

## Conclusions

A patient with RA who developed pelvic peritonitis during administration of biologics and immunosuppressive agents was described. Before starting such agents, patients must be questioned regarding the presence of an intrauterine foreign body.
